# Supplementary figures and images for: CytoPy: An autonomous cytometry analysis framework
Source: PLoS Comput Biol. 2021 Jun 8;17(6):e1009071. doi: 10.1371/journal.pcbi.1009071 (PMC8213167; doi:10.1371/journal.pcbi.1009071)

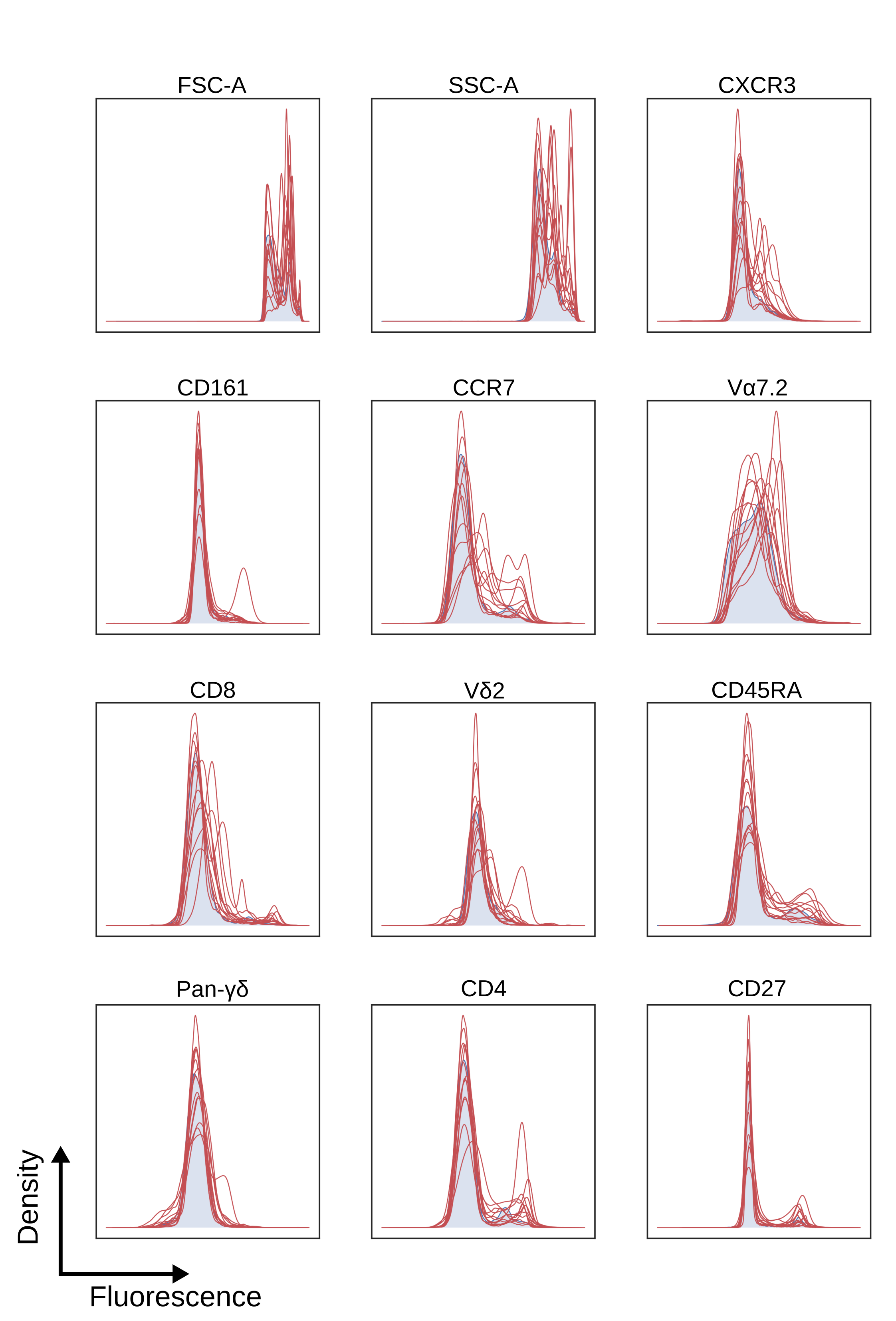

Supplement: S1 Fig — (TIFF) [file pcbi.1009071.s001.tiff]

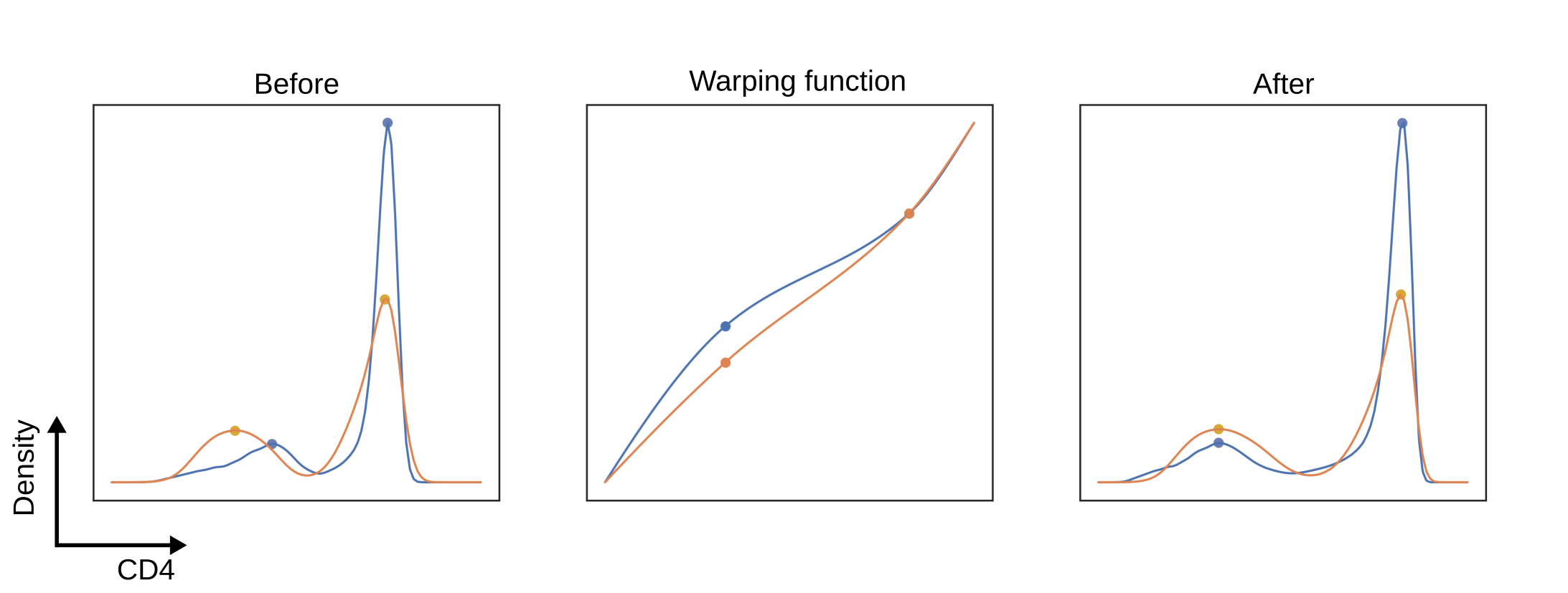

Supplement: S2 Fig — Left, target (orange) PDF compared to the reference (blue) prior to alignment. Centre, warping function defined between landmarks by taking a monotone cubic interpolation. Right, registered curve with aligned peaks obtained using function composition. (TIFF) [file pcbi.1009071.s002.tiff]

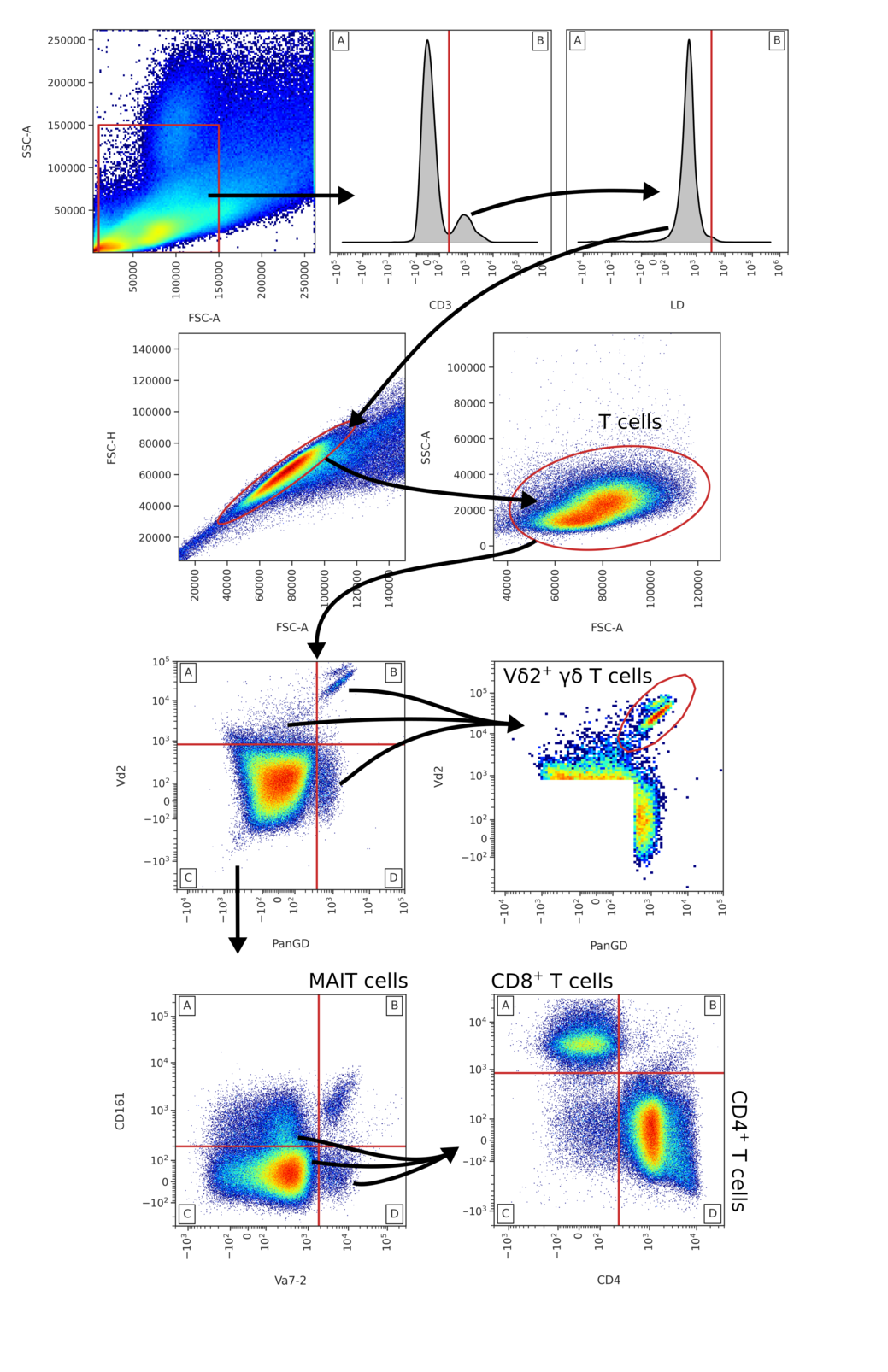

Supplement: S3 Fig — (TIFF) [file pcbi.1009071.s003.tiff]

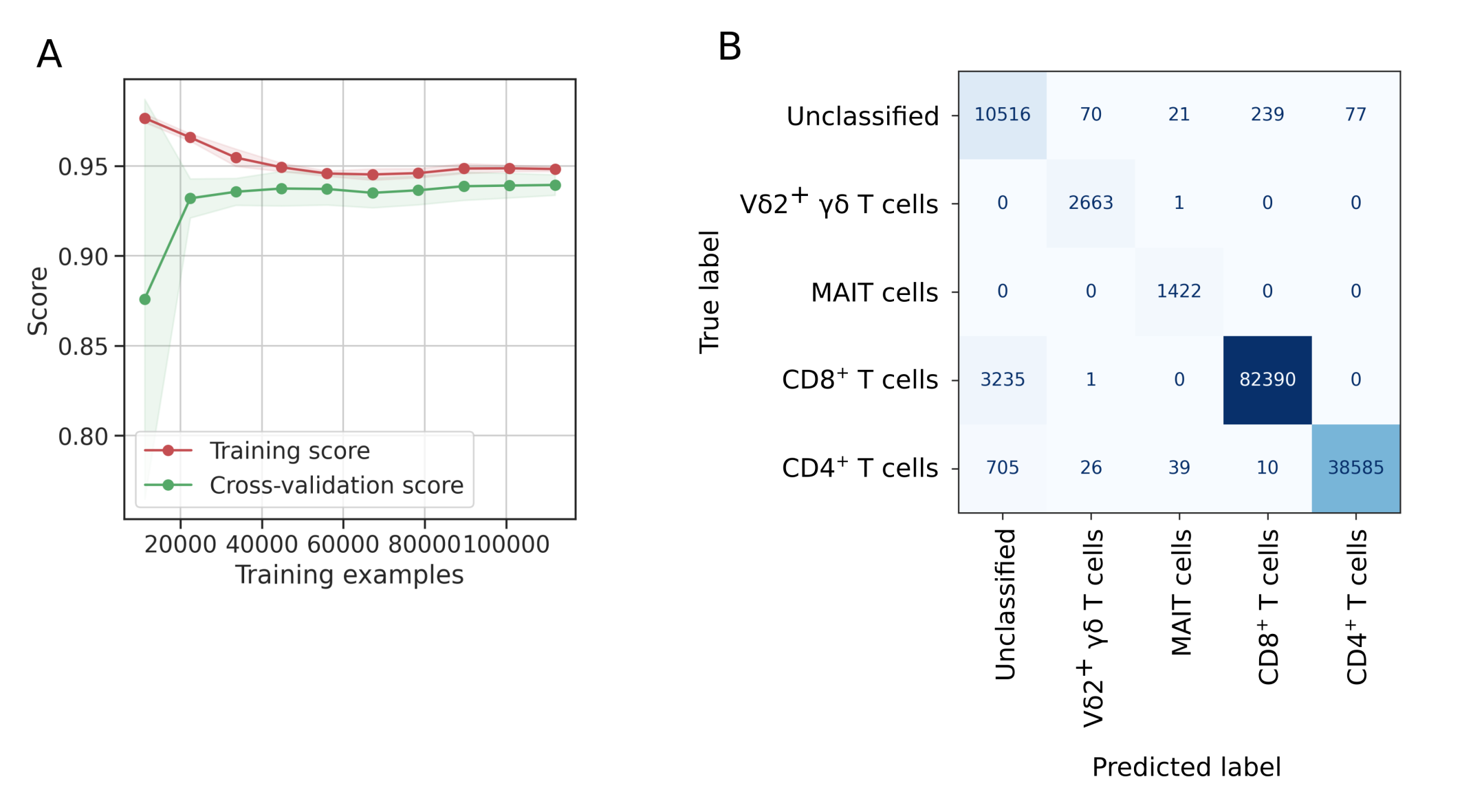

Supplement: S4 Fig — Example of a learning curve (A) for training XGBoost for identifying T cells subsets, and confusion matrix (B) for the same algorithm when exposed to validation data. (TIFF) [file pcbi.1009071.s004.tiff]

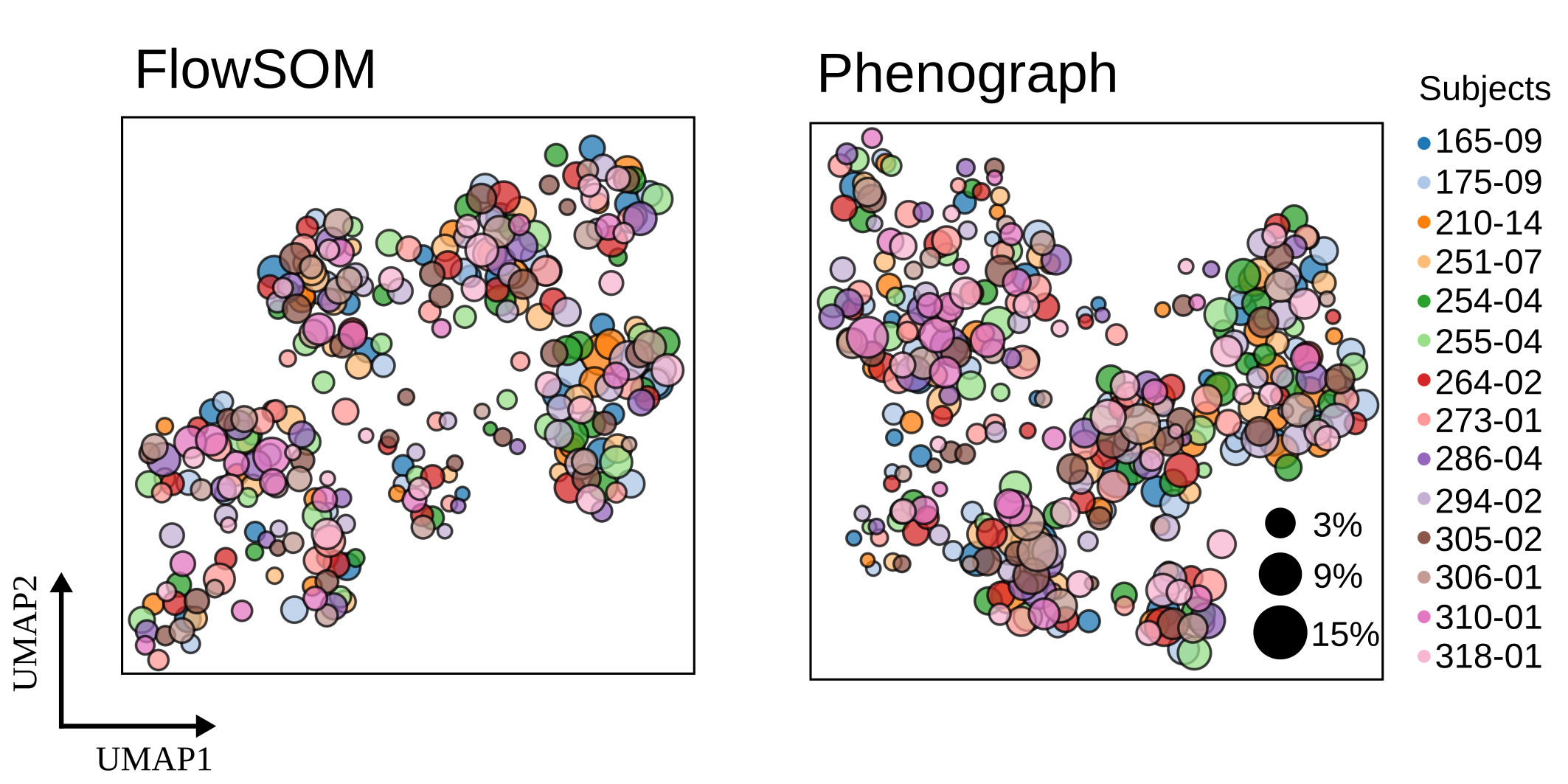

Supplement: S5 Fig — The size of the data points correspond to the % of T cells the given cluster represents from the respective patient. Subject numbers shown are unique patient sample identifiers. (TIFF) [file pcbi.1009071.s005.tiff]

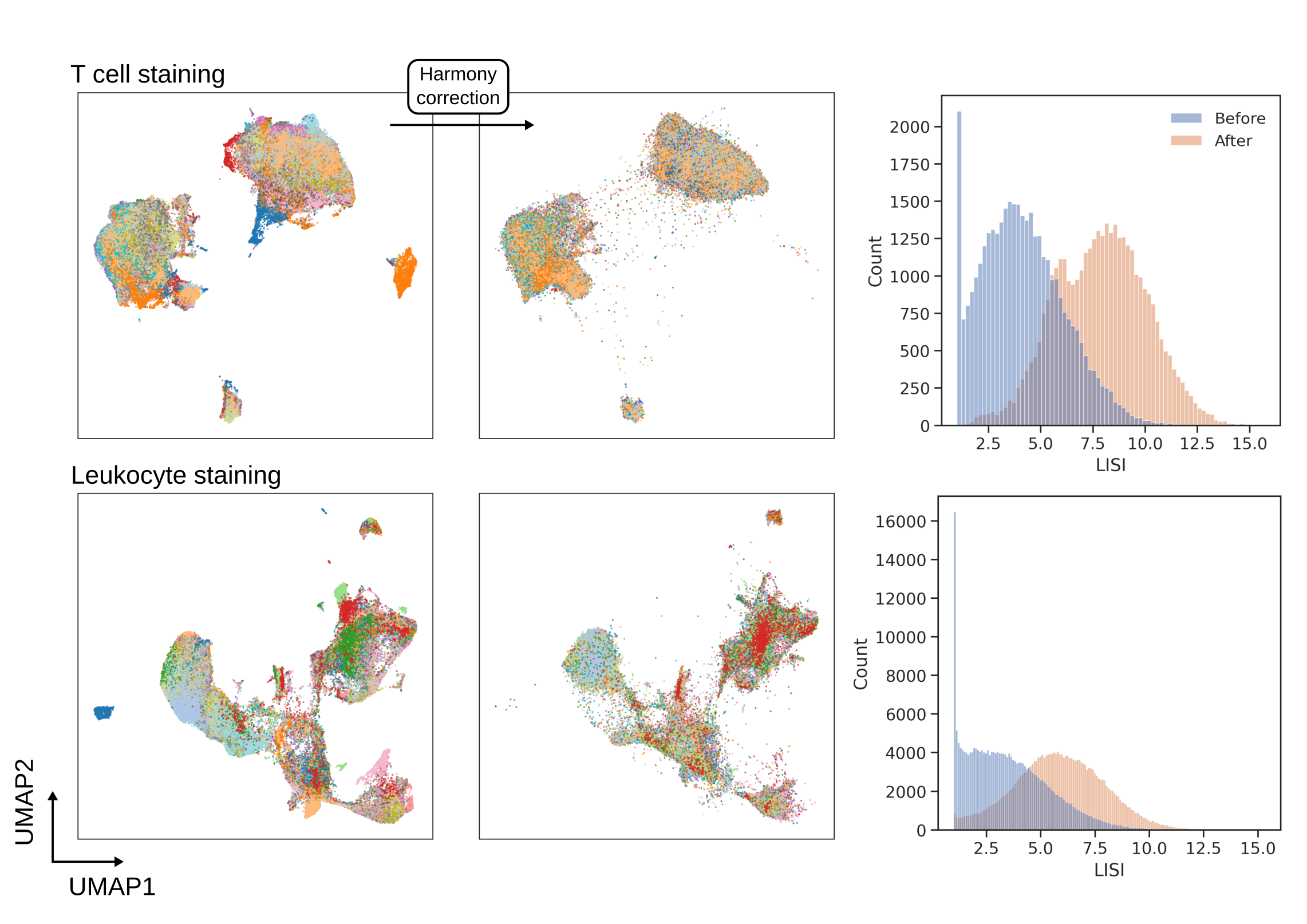

Supplement: S6 Fig — Single cell UMAP plots are coloured to show the origin of cells where each colour is a unique patient. UMAP plots following correction and LISI distribution show the effectiveness of Harmony to correct for technical variation. (TIFF) [file pcbi.1009071.s006.tiff]

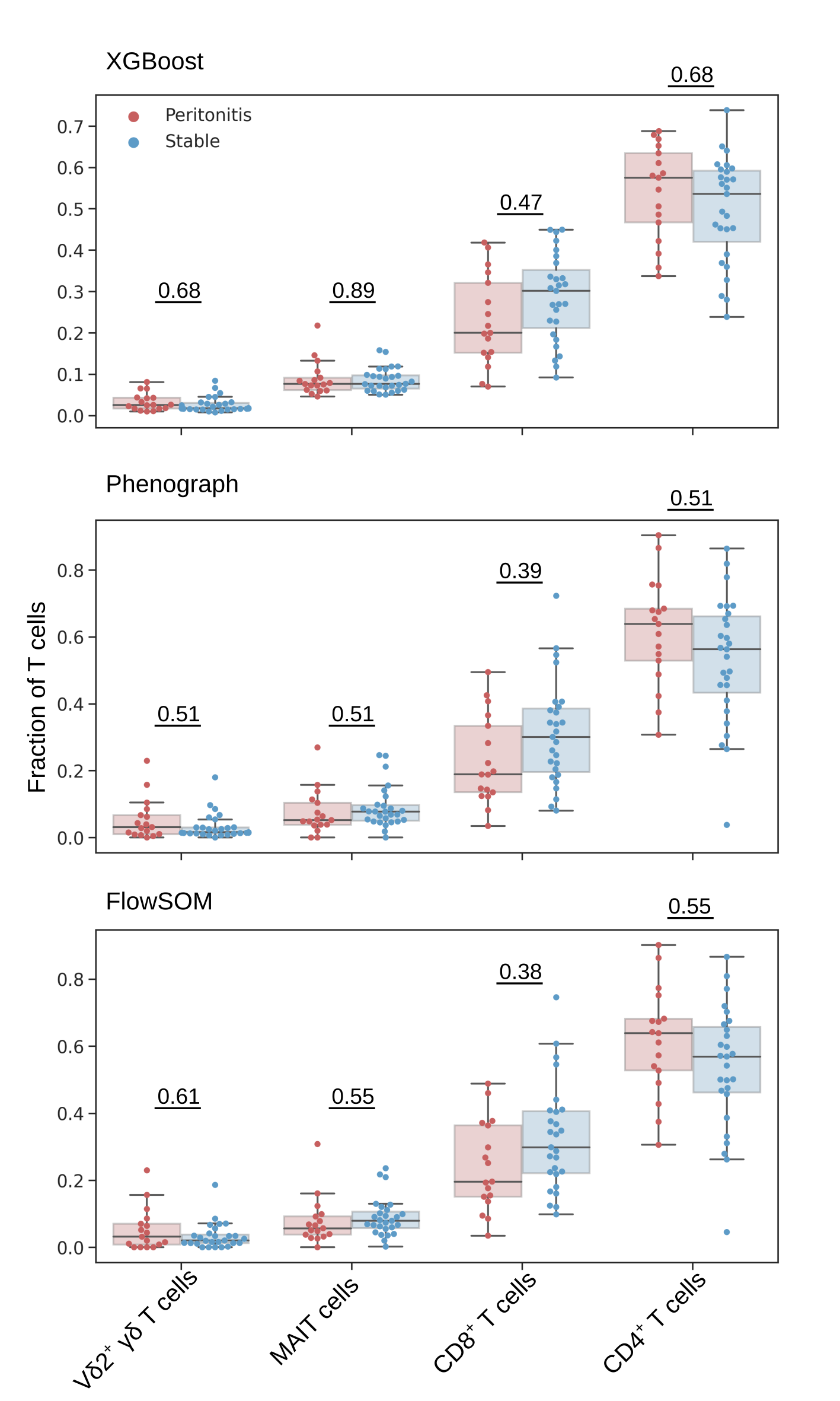

Supplement: S7 Fig — Mann-Whitney U tests were applied for comparisons between patients with acute peritonitis and stable controls and p-values are reported after correction for multiple comparisons using Holm’s method (significance level was set as 0.05). (TIFF) [file pcbi.1009071.s007.tiff]
