# Supplementary material for: CytoPy: An autonomous cytometry analysis framework
Source: PLoS Comput Biol. 2021 Jun 8;17(6):e1009071. doi: 10.1371/journal.pcbi.1009071 (PMC8213167; doi:10.1371/journal.pcbi.1009071)
Supplement: S1 Table — *Performance from the original competition as reported by Aghaeepour N et al. [1]; all other algorithms are implemented through CytoPy. (DOCX) [file pcbi.1009071.s008.docx]

| Classifier | GvHD | DLBCL | HSCT | WNV | ND | Mean |
| --- | --- | --- | --- | --- | --- | --- |
| RadialSVM* | 0.89 (0.83, 0.95) | 0.84 (0.80, 0.87) | 0.98 (0.96, 0.99) | 0.96 (0.94, 0.97) | 0.93 (0.92, 0.94) | 0.92 |
| flowClust/Merge* | 0.92 (0.88, 0.95) | 0.92 (0.89, 0.94) | 0.95 (0.92, 0.97) | 0.84 (0.82, 0.86) | 0.89 (0.88, 0.90) | 0.90 |
| randomForests* | 0.85 (0.78, 0.91) | 0.78 (0.74, 0.83) | 0.81 (0.79, 0.83) | 0.87 (0.84, 0.90) | 0.94 (0.92, 0.95) | 0.85 |
| FLOCK* | 0.82 (0.77, 0.87) | 0.91 (0.89, 0.93) | 0.86 (0.76, 0.93) | 0.86 (0.82, 0.89) | 0.86 (0.77, 0.92) | 0.86 |
| CDP* | 0.78 (0.68, 0.87) | 0.95 (0.93, 0.97) | 0.75 (0.71, 0.78) | 0.86 (0.84, 0.88) | 0.83 (0.80, 0.86) | 0.80 |
| Ensemble clustering* | 0.91 | 0.94 | 0.95 | 0.92 | 0.94 | 0.93 |
| Logistic regression | 0.93 (0.92, 0.95) | 0.94 (0.93, 0.95) | 0.97 (0.96, 0.97) | 0.91 (0.90, 0.92) | 0.82 (0.81, 0.83) | 0.91 |
| Linear discriminant analysis | 0.94 (0.92, 0.97) | 0.98 (0.97, 0.98) | 0.95 (0.93, 0.97) | 0.92 (0.9, 0.94) | 0.81 (0.80, 0.82) | 0.92 |
| Radial SVM | 0.97 (0.96, 0.98) | 0.98 (0.97, 0.98) | 0.97 (0.96, 0.97) | 0.97 (0.96, 0.97 | 0.91 (0.90, 0.91) | 0.96 |
| K nearest neighbours | 0.95 (0.93, 0.97) | 0.97 (0.96, 0.98) | 0.94 (0.93, 0.96) | 0.95 (0.94, 0.95) | 0.89 (0.89, 0.90) | 0.94 |
| XGBoost | 0.99 (0.98, 0.99) | 0.98 (0.97, 0.98) | 0.99 (0.99, 0.99) | 0.99 (0.98, 0.99) | 0.99 (0.98, 0.99) | 0.99 |
| Feed-forward deep neural net | 0.96 (0.93, 0.97) | 0.93 (0.91, 0.95) | 0.97 (0.96, 0.98) | 0.98 (0.98, 0.99) | 0.92 (0.92, 0.93) | 0.95 |

**S1 Table.** Performance of supervised classification algorithms for identifying cell populations from the FlowCAP competition data. *Performance from the original competition as reported by Aghaeepour N *et al.* [1]; all other algorithms are implemented through CytoPy.

**References**

1. Aghaeepour N, Finak G, Hoos H, Mosmann TR, Brinkman R, Gottardo R, et al. Critical assessment of automated flow cytometry data analysis techniques. Nat Methods. 2013;10(3):228–38.
