# Supplementary material for: CytoPy: An autonomous cytometry analysis framework
Source: PLoS Comput Biol. 2021 Jun 8;17(6):e1009071. doi: 10.1371/journal.pcbi.1009071 (PMC8213167; doi:10.1371/journal.pcbi.1009071)
Supplement: S2 Table — (DOCX) [file pcbi.1009071.s009.docx]

| **Culture result** | ***n*** |
| --- | --- |
| Coagulase-negative *Staphylococcus* | 6 |
| Alpha-haemolytic *Streptococcus* | 3 |
| *Staphylococcus aureus* | 1 |
| *Escherichia coli* | 1 |
| *Streptococcus agalactiae* | 1 |
| *Corynebacterium amycolatum* | 1 |
| *Pseudomonas aeruginosa* | 1 |
| Yeast | 1 |
| Mixed growth | 2 |
| No growth/unknown | 4 |

**S2 Table**. Summary of microbiological culture results for peritoneal dialysis patients with acute peritonitis
