# Supplementary material for: CytoPy: An autonomous cytometry analysis framework
Source: PLoS Comput Biol. 2021 Jun 8;17(6):e1009071. doi: 10.1371/journal.pcbi.1009071 (PMC8213167; doi:10.1371/journal.pcbi.1009071)
Supplement: S3 Table — (DOCX) [file pcbi.1009071.s010.docx]

| Marker | Fluorochrome | Manufacturer (Clone) |
| --- | --- | --- |
| CD3 | APC/Fire | BioLegend (UCHT1) |
| CD4 | PE-Cy5.5 | BioLegend (OKT4) |
| CD8 | Brilliant Violet 711 | BioLegend (RPA-T8) |
| CD161 | APC | Miltenyi Biotec (191B8) |
| Vα7.2 | Brilliant Violet 605 | Biolegend (3C10) |
| TCR-pan-γδ | PE-Cy5 | Beckman Coulter (IM2662) |
| Vδ2 | PE | BD Biosciences (B6 RUO) |
| CCR7 | Brilliant Violet 421 | BioLegend (G043H7) |
| CD27 | PE-Cy7 | BioLegend (M-T271) |
| CD45RA | PE Dazzle | BioLegend (HI100) |

**S3 Table.** Staining panel for T cells
