# Supplementary material for: CytoPy: An autonomous cytometry analysis framework
Source: PLoS Comput Biol. 2021 Jun 8;17(6):e1009071. doi: 10.1371/journal.pcbi.1009071 (PMC8213167; doi:10.1371/journal.pcbi.1009071)
Supplement: S4 Table — (DOCX) [file pcbi.1009071.s011.docx]

| Marker/Cytokine | Fluorochrome | Manufacturer (Clone) |
| --- | --- | --- |
| CD45 | Alexa Fluor 700 | BioLegend (2D1) |
| CD14 | FITC | BioLegend (63D3) |
| CD16 | Per-CP Cy5.5 | BioLegend (3G8) |
| CD3 | APC/Fire | BioLegend (UCHT1) |
| SIGLEC-8 | APC | BioLegend (7C9) |
| CD1c | Brilliant Violet 421 | BioLegend (L161) |
| CD15 | Brilliant Violet 605 | BioLegend (SSEA-1) |
| HLA-DR | Brilliant Violet 711 | BioLegend (L243) |
| CD116 | PE | BioLegend (4H1) |
| CD19 | PE-Cy7 | BioLegend (HIB19) |

**S4 Table.** Staining panel for leukocytes
