# Supplementary material for: CytoPy: An autonomous cytometry analysis framework
Source: PLoS Comput Biol. 2021 Jun 8;17(6):e1009071. doi: 10.1371/journal.pcbi.1009071 (PMC8213167; doi:10.1371/journal.pcbi.1009071)
Supplement: S1 Methods — (DOCX) [file pcbi.1009071.s012.docx]

**Supplementary Methods**

**1. Patients**

The study cohort comprised 21 adult individuals receiving peritoneal dialysis (PD) who were admitted between October 2016 and October 2018 to the University Hospital of Wales, Cardiff, on day 1 of acute peritonitis, before commencing antibiotic treatment (47.6% female; median age 53.0 years, range 30.0-86.0 years). 30 age and gender-matched individuals receiving PD and with no previous infections for at least 3 months served as stable, non-infected controls (53.3% female; median age 59.7 years, range 39.7-84.3 years). Subjects known to be positive for HIV or hepatitis C virus were excluded. Clinical diagnosis of acute peritonitis was based on the presence of abdominal pain and cloudy peritoneal effluent with >100 white blood cells/mm^3^. According to the microbiological analysis of the effluent by the routine Microbiology Laboratory, Public Health Wales, episodes of peritonitis were defined as infections caused by Gram-positive or Gram-negative organisms. Cases of fungal infection and negative or unclear culture results were excluded from this analysis. A summary of the bacterial culture results for patients with peritonitis are shown in S2 Table.

**2. FlowCAP**

Supervised classifiers in CytoPy were compared using data provided in the Flow Cytometry: Critical Assessment of Population Identification Methods (FlowCAP) challenge [1], where the challenge is to accurately separate cells into subsets based on single cell phenotype. The FlowCAP-I data consist of four human studies (graft-versus-host disease, diffuse large B-cell lymphoma, symptomatic West Nile virus infection, and healthy donors) and one mouse study (hematopoietic stem cell transplant). Data were labelled and pre-processing performed (removal of debris, dead material, and with fluorescence compensation applied) at source by the laboratory responsible for acquiring the original data. Here, classifiers were trained on 25% of data and classification performance tested on the remaining 75%. Performance was reported as the average of weighted F1 scores across all five datasets, where the F1 score for data with |*C*| set of possible classes is given as:

$$weightedF1score=\frac{2}{\left| C \right|}\sum_{c\in C} \frac{{precision}_{c}\cdot{recall}_{c}}{{precision}_{c}+{recall}_{c}}$$

Six supervised machine learning algorithms, housed within CytoPy, were compared without hyperparameter tuning:

1. Logistic regression with balanced class-weights; implemented in Scikit-Learn version 0.24
2. Linear discriminant analysis without any shrinkage and number of components equal to either the number of classes or number of features, depending on which is minimum; implemented in Scikit-Learn version 0.24
3. Support vector machine with a radial basis function kernel without regularisation and γ as $\frac{1}{n}$where *n* is the number of available features; implemented in Scikit-Learn version 0.24
4. K nearest neighbours classifier with *k* equal to 30; implemented in Scikit-Learn version 0.24
5. XGBoost using default parameters; implemented in xgboost version 1.2
6. Feed-forward neural network with three hidden layers of size 12, 6, and 3 nodes, L2 penalty of 1×10^−4^, softplus activation function on the hidden layers, softmax activation function of the outer most layer, and categorical cross-entropy as the loss function; implemented in Tensorflow Keras version 2.4

**3. Flow cytometry**

Peritoneal leukocytes were harvested from overnight dwell effluents and processed as described previously [2,3]; samples were treated with DNase (Sigma; 1:2,500 dilution) when excessive debris was visually apparent. Leukocyte populations in total effluent were stained using monoclonal antibodies against CD1c, CD3, CD14, CD15, CD16, CD19, CD45, CD116, HLA-DR and Siglec-8 (S3 Table) and identified as CD45^+^ immune cells, CD3^+^ T cells, CD19^+^ B cells, CD15^−^CD14^+^ monocytes/macrophages, CD15^+^ neutrophils, CD15^−^CD14^+/−^CD1c^+^ dendritic cells, and CD15^−^SIGLEC-8^+^ eosinophils. T cell subsets in peripheral blood mononuclear cells (PBMCs) and in peritoneal effluent were stained after Ficoll (Ficoll-Paque PLUS; Fisher Scientific) separation of blood and peritoneal leukocytes, respectively, using monoclonal antibodies against CD3, CD4, CD8, CD161, TCR-Vα7.2, TCR-Vδ2, TCR-pan-γδ, CD45RA, CCR7 and CD27 (S4 Table). Cell acquisition by flow cytometry was performed using a 16 colour BD LSR Fortessa cell analyser (BD Biosciences). Live single cells were gated based on side and forward scatter area/height and live/dead staining (fixable Aqua; Invitrogen).

**4. Autonomous gating**

Autonomous gates inherit from the parent class *Gate* (providing access to common utilities such as data transformations) but are divided into the following classes to facilitate gating geometries:

1. The ***ThresholdGate*** divides data in one or two-dimensional space using a threshold of positivity (a straight line that divides data into positive and negative regions). Thresholds are found as regions of minimal density in the estimated probability density function of the observed data; estimated with a fast convolution-based kernel density estimation algorithm [4] with a Gaussian kernel and bandwidth estimated using the Silverman method.
2. The ***PolygonGate*** allows the user to apply any Scikit-Learn clustering algorithm to two-dimensional data; including the popular HDBSCAN algorithm [5]. Polygon gates are generated from the resulting clusters by computing their convex hull, the contents of this polygon are used to construct *Population* objects.
3. The ***EllipseGate*** allows the user to apply the probabilistic mixture model algorithms of the Scikit-Learn library to generate elliptical gates. For each component of the mixture model the covariance matrix is used to generate a confidence ellipse, surrounding data and emulating a gate. The ellipse is centred on the mean of the chosen component and oriented in the direction of the first eigenvector of the covariance matrix. The approximate likelihood of a data point falling within the bounds of the ellipse can be estimated using the chi-squared distribution. A hyperparameter, ‘conf’, is provided (default is 0.95) as the percentile of the chi-squared distribution to generate an ellipse where the length of the primary axis (the longest axis) is such that the chosen percentage of data attributed to this component is contained within the ellipse. This elliptical gate is then committed to the database as a polygon object.

**5. Manual gating**

T cells from whole blood were manually gated in FlowJo v10.7 (TreeStar) by two independent experts. The total number of events for each gate of interest were exported as a CSV file. The average number of events between the two independent analysts was used for comparison of automated methods to manual gating.

**6. References**

1. Aghaeepour N, Chattopadhyay P, Chikina M, Dhaene T, Van Gassen S, Kursa M, et al. A benchmark for evaluation of algorithms for identification of cellular correlates of clinical outcomes. Cytom Part A. 2016;89(1):16–21.
2. Zhang J, Friberg IM, Kift-Morgan A, Parekh G, Morgan MP, Liuzzi AR, et al. Machine-learning algorithms define pathogen-specific local immune fingerprints in peritoneal dialysis patients with bacterial infections. Kidney Int. 2017;92(1):179–91.
3. Lin CY, Roberts GW, Kift-Morgan A, Donovan KL, Topley N, Eberl M. Pathogen-specific local immune fingerprints diagnose bacterial infection in peritoneal dialysis patients. J Am Soc Nephrol. 2013;24(12):2002–9.
4. Odland T. KDEpy: Kernel Density Estimation in Python. Zenodo; 2018. Available from: <http://doi.org/10.5281/zenodo.2392268>
5. McInnes L, Healy J, Astels S. hdbscan: Hierarchical density based clustering. J Open Source Softw. 2017;2(11):205.
